# Evaluation of antibody response against recombinant domain III proteins of dengue virus type 1 and 2

**DOI:** 10.3934/microbiol.2017.2.248

**Published:** 2017-04-14

**Authors:** Nagesh K Tripathi, Ambuj Shrivastava

**Affiliations:** Bioprocess Scale up Facility, Defence Research and Development Establishment, Jhansi Road, Gwalior-474002, India

**Keywords:** fermentation, scale up, purification, vaccine, ELISA, immunogenicity

## Abstract

Dengue, a mosquito borne viral disease caused by dengue virus has emerged as a major health problem during the last few decades. The envelope domain III (DIII) protein of dengue virus is highly immunogenic and capable of inducing neutralizing antibodies against wild-type dengue virus. The envelope domain III protein is a potential subunit vaccine candidate as well as a diagnostic reagent for dengue. This report describes the high yield production and immunogenicity of recombinant DIII proteins of dengue virus type 1 and 2. The subunit DIII proteins were produced in *Escherichia coli* using batch and fed-batch fermentation process. Immobilized metal affinity chromatography was used to capture DIII proteins of dengue virus type 1 and 2. The purified proteins were refolded by diafiltration to achieve biologically active proteins. After fed-batch fermentation, the recombinant *E. coli* resulted in purified DIII proteins of about 10.06 mg and 47.70 mg per gram of dry cell weight for recombinant dengue virus type 1 and 2 respectively with more than 95% purity. Biological function of the purified DIII proteins were confirmed by their ability to generate DIII specific antibodies in mice. The DIII antigens in combination with adjuvant resulted antibody endpoint titers of 1:64,000 and 1:1,28,000 for recombinant dengue virus type 1 and 2 respectively. These findings establish that the DIII proteins in combination with adjuvant are immunogenic, which suggests that refolded and purified DIII proteins can be a potential vaccine candidates.

## 1. Introduction

Dengue is an important public health problem of people living in the tropical and subtropical areas. Dengue virus, which comprises four antigenically distinct serotypes (1–4), belongs to the *Flavivirus* genus in the *Flaviviridae* family. Dengue virus causes a wide range of diseases ranging from mild dengue fever to severe dengue hemorrhagic fever and dengue shock syndrome [1],[2]. Vaccination is a cost-effective way to control the threat of infectious diseases. In the past six decades, effort has been made to develop a vaccine against dengue virus infection [1],[3],[4]. Dengue virus is a single stranded RNA virus. The genome of dengue virus encodes three structural proteins (capsid protein, pre-membrane protein and envelope protein) and seven non-structural proteins. The envelope protein has three domains (domain I, domain II and domain III). The domain III contains approximately hundred amino acid residues stabilized by single disulfide bond. Domain III is an immunoglobulin like domain and responsible for receptor binding, contains multiple type and sub-type specific neutralizing epitopes [4]–[7]. Experimental evidence has demonstrated that the domain III protein is highly immunogenic and elicit the production of neutralizing antibodies against wild-type dengue virus [3],[4],[8],[9]. These characteristics of domain III protein indicate that it would be an important immunogen for the development of a possible dengue vaccine candidate and also a potential diagnostic reagent for improved clinical diagnosis of dengue virus infection [3]–[6],[7],[9],[10]. In order to facilitate further study with domain III protein, there is a necessity to develop a large scale processes that achieve this protein in large amount with biologically active form.

Most of the recombinant proteins are expressed either in bacteria, yeasts, insect cell lines or transgenic plant cells. However, *E. coli* a bacterial expression host is still a choice of system due to its well characterized genetics, rapid growth rate and use of inexpensive substrates for cultivations [11],[12],[13]. Many proteins of vaccine or therapeutic use are produced in *E. coli* using batch and fed-batch fermentation process [11]–[14]. It would be very useful if the optimized production as well as purification processes could be easily scaled up to an industrial scale for the bioprocess industry. Fermentation media, mode of fermentation, scale up and culture conditions are the most significant factors in a production process. Media composition especially with the essential carbon and nitrogen components is optimized to produce large amount of desired product. Fed-batch fermentation is a common technique in this regard to increase the productivity of recombinant proteins [14],[15],[16]. Increasing the scale of production is usually associated with reduction in the desired product yield due to the complexity associated with fermentation process. Therefore, it is of great importance to study the scaling up of laboratory scale fermentation process and apply suitable strategy in order to enhance the productivity of the desired recombinant protein on the large scale.

High-level expression of recombinant proteins in *Escherichia coli* often accumulates as insoluble, inactive aggregates in the form of inclusion bodies *in vivo* [17],[18],[19]. Although the recombinant protein expression in the form of IBs has advantages, the inactive recombinant proteins need to be converted into soluble and correctly folded proteins [20]. The solubilization of IBs was carried out using higher concentration of denaturing agent which must be removed using refolding processes to obtain biologically active recombinant product for vaccine studies. In recent years, several refolding techniques such as rapid dilution, dialysis, diafiltration, high pH solubilization and on column refolding using chromatographic techniques have been used for recovery of bioactive recombinant proteins [17],[20],[21],[22]. Immobilized metal affinity chromatography (IMAC) has emerged as a powerful technique for purification of recombinant proteins [22]. A biospecific ligand covalently attached affinity matrix is required in IMAC. IMAC has good selectivity, ability to perform protein refolding and potential to achieve more than 95% purity in single step. In the present study, we propose an IMAC based one step purification strategy for DIII proteins of dengue virus type 1 and 2 and renaturation by diafiltration to recover the biologically active protein. The objective of this study was to produce *E. coli* derived biologically active DIII proteins of dengue virus type 1 and 2 at large scale for dengue vaccine development. Here, we report on the large scale production, purification and characterization of recombinant DIII proteins of dengue virus type 1 and 2. Further we have also demonstrated the immunogenic potential of purified and refolded DIII proteins for dengue vaccine development.

## 2. Materials and Methods

### 2.1. Reagents, media and chemicals

Media used for shake flask culture and fermentations were from BD (Difco), USA. Chemicals for sodium dodecyl sulphate polyacrylamide gel electrophoresis (SDS-PAGE) were from Bio-Rad, USA. Protein molecular weight markers were from Thermofisher, USA. Chromatography resin and pre-packed columns used for purifications were from GE Healthcare, Sweden. Antibiotics, Isopropyl β-D-thiogalactoside (IPTG), secondary antibody and all other analytical and molecular biology grade chemicals were from Sigma, USA. Microtiter plates were from Nunc, Germany.

### 2.2. Expression constructs

*E. coli* strain BLR (DE3) harboring a pET30a+ based expression vector (Novagen, USA) was used for recombinant envelope domain III protein production of dengue virus type 1 and 2. This vector contains a strong phage T7 promoter bearing 6× histidine tag and synthesized DIII genes inserted into pET30a+ vector through *Nde*I and *Sal*I restriction endonucleases sites for dengue virus type 1 and 2. The accession number for gene sequence of dengue virus type 1 and 2 were AY422786 and AF410376 respectively. Stock cultures of the microorganisms were maintained in 30% glycerol (v/v) at –80 °C.

### 2.3. Inocula preparation

Erlenmeyer shake flasks of 250 ml capacity containing 50 ml of LB medium (Tryptone, 10.0 g; Yeast extract, 5.0 g; NaCl, 5.0 g per liter) with antibiotics (50 µg/ml kanamycin and 12.5 µg/ml tetracycline) were inoculated with a cryovial of stock cultures containing DIII gene of dengue virus type 1 and 2 from the frozen cell stock. The seeded cultures were incubated at 180 rpm and 37 °C for 8 h in an incubator-shaker. Primary seed culture (1% v/v) was used to inoculate 100 ml of LB and modified SB medium (Di-potassium phosphate, 11.4 g; Sodium chloride, 0.5 g; Ammonium chloride, 1.0 g, Mono-potassium phosphate, 2.2 g; Magnesium sulphate, 2.4 g; yeast extract, 24 g; tryptone, 12 g and glycerol 20 ml per liter) in 500 ml Erlenmeyer shake flasks and incubated for overnight at 37 °C and 180 rpm. For bioreactor inoculation, overnight grown culture as above in two liter shake flasks containing 500 ml of modified SB medium was used.

### 2.4. Shake flask cultivations

For shake flask expressions, one liter Erlenmeyer flasks containing 200 ml of LB and modified SB medium with respective antibiotics were inoculated with 2% (v/v) of overnight grown secondary seed cultures in respective media for recombinant dengue virus type 1 (rDen1 DIII) and recombinant dengue virus 2 (rDen2 DIII) proteins separately. The cultures were incubated at 37 °C and 200 rpm in an Incubator shaker. When the cultures reach at mid exponential phase (OD: 0.6–1.0), inducer (1 mM IPTG) was added and induction was allowed to proceed for another 4 h to express proteins. The samples were taken to measure OD at 600 nm and dry cell weight. Cultures were centrifuged to recover pellet at 6000 rpm for 20 min at 4 °C to analyze rDen1 EDIII and rDen2 DIII proteins expressions.

### 2.5. Laboratory scale batch bioprocesses

Bioreactor of 5 liter capacity (New Brunswick, USA) was used for production of rDen1 DIII and rDen2 DIII proteins using batch process. Bioreactor vessel containing 4 liter of modified SB medium with respective antibiotics was inoculated with 5% (v/v) of overnight grown cultures. The cultivation temperature was maintained constant at 37 °C. Aeration rate was 0.5 to 2.0 vvm during cultivation processes. The dissolved oxygen (DO) concentration was maintained at 20–30% of air saturation by varying agitation rate between 200 and 500 rpm as well as using pure oxygen with air, if necessary. The cultivation pH was controlled between 6.8 and 7.0 using 25% ammonia solution or 1 N HCl whenever required. Antifoam was added whenever required to prevent foaming during cultivation. The cultures were grown till mid log phase (4.5–5.5 h post inoculation), after which inducer (1 mM IPTG) was added for rDen1 DIII and rDen2 DIII protein expressions. Cells were recovered after four hours of post induction using centrifugation at 6000 rpm for 30 min at 4 °C. The sampling was performed at regular interval to check culture growth by measuring OD_600_.

### 2.6. Laboratory scale fed-batch bioprocesses

Bioreactor of 10 liter capacity (New Brunswick, USA) was used for fed-batch cultivations to produce rDen1 DIII and rDen2 DIII proteins. Fermentation conditions were controlled as described for the batch cultivations. About 400 ml of overnight grown culture was inoculated into bioreactor vessel containing 8 liter of modified SB medium in presence of respective antibiotics. Dissolved oxygen was controlled using agitation between 100 and 800 rpm, aeration between flow rate of 0.5 to 1.5 vvm of air and with pure oxygen as required at high cell densities. At the middle of the logarithmic phase when carbon source was consumed from the culture media, indicated by increase in dissolved oxygen and pH, feeding of nutrient was started. At this point (∼5.5 hours of post inoculation), feeding was initiated with feed medium. The feed medium per liter contained glycerol (400 ml), yeast extract (300 g) and magnesium sulphate (10 g) supplemented with antibiotics. Feeding medium was pumped to the bioreactor vessel in a linearly increasing manner from one milliliter to two milliliter per minute over an eight hours period. If the dissolved oxygen concentration levels decreased below set value (30%), the pump was stopped till DO levels returned to set value. Similarly if the pH value decreased below set value (6.8), the pump was stopped till pH value returned to set value. A combination of pH stat and DO stat feedback mechanism regulated the feed rate by feeding in a control volume of feed in response to increase in pH and DO of culture. Inducer (1 mM IPTG) was added to initiate DIII protein expression and cells were further continued to grow for another 4 h before termination of fermentation processes. Measurement of OD was done on hourly basis to check the growth of culture.

### 2.7. Laboratory scale purifications and refolding

For purifications of rDen1 DIII and rDen 2 DIII proteins at small scale, cells were harvested using centrifugation at 6000 rpm for 30 min at 4 °C and then washed by cell wash buffer (10 mM Tris-HCl pH 8.0, 10 mM EDTA, 100 mM NaCl). The washed cells were resuspended in cell lysis buffer (10 mM Tris-HCl pH 7.5, 5 mM EDTA, 100 mM NaCl, 100 µg/ml of Lysozyme and 1 mM phenylmethylsulfonyl ﬂuoride-PMSF) and lysed by sonication using a sonicator (Sonics, USA) at a setting of 70% frequency using high gain probe. The sonicator was programmed to provide 9 s pulses with 9 s pause for a total period of 10 min. The inclusion bodies (IBs) were recovered by centrifugation of lysate at 10,000 rpm for 30 min at 4 °C. The recovered IBs were washed by IBs wash buffer 1 (50 mM NaH_2_PO_4_ pH 6.0, 5 mM EDTA, 200 mM NaCl, 0.5 M urea, 1% TritonX-100) and IBs wash buffer 2 (50 mM NaH_2_PO_4_ pH 6.0, 1 mM EDTA and 1 M NaCl) to remove impurities. The washed IBs were solubilized in IBs solubilization buffer (10 mM Tris-HCl, pH 8.0, 100 mM NaH_2_PO_4_, 100 mM NaCl, 8 M Urea) and further centrifuged at 10,000 rpm for 40 min at 4 °C. The supernatant were removed and further filtered using 0.45 µm filter. The small scale chromatographic purifications were performed on an AKTA Explorer Fast Protein Liquid Chromatography system (GE Healthcare, Sweden) at room temperature. The clarified supernatant was loaded onto a 20 ml Hisprep 16/10 immobilized metal affinity chromatography column. The column was pre-equilibrated with equilibration buffer (10 mM Tris HCl, 100 mM NaCl, 100 mM NaH_2_PO_4_, 8 M Urea, pH 8.0). The linear flow rate used for this column was 150 cm/h. The column was washed with ten column volume of wash buffer (equilibration buffer with pH 6.3). The protein was eluted using elution buffer (equilibration buffer with pH 4.3). The eluted fractions were pooled and refolded by diafiltration using centrifugal devices with diafiltration buffer (50 mM phosphate buffer, 250 mM NaCl, pH 5.8) containing progressively decreasing urea concentration (6 M, 4 M, 2 M, 1 M and 0 M). The refolded rDen1 DIII and rDen2 DIII proteins were further used for determination of biological activity.

### 2.8. Scale up of fermentation process

The scale up of recombinant dengue virus type 2 domain III protein production was also carried out similar to the process described in our earlier study [23] in a 100 liter bioreactor (New Brunswick, USA). Briefly, the secondary seed culture as grown above was inoculated into 5 liter bioreactor vessel containing 4 liters of modified SB medium. This bioreactor culture was cultivated about 6 hours at conditions described in batch bioprocess section. The seed culture (4 liter) obtained from 5 liter bioreactor was transferred to the 100 liter bioreactor containing 80 liter of modified SB medium. Aeration and agitation were varied in the range of 0.25–0.6 vvm and 50–300 rpm respectively. The cultivation temperature and initial pH was 37 °C and 7.0 respectively. Automatic addition of ammonia and phosphoric acid were used to maintain pH between 6.8 and 7.0. The dissolved oxygen, level was maintained at 20–30% saturation. Anti-foam was added to prevent foaming whenever needed. At mid exponential phase, about 5.5 hours of post inoculation, inducer was added into the bioreactor culture at a concentration of 1 mM and further grown for 4 hours before harvesting. OD_600_, wet and dry cell weight were analyzed during fermentation process.

### 2.9. Pilot scale purification and refolding

Fermentation broth obtained from pilot scale fermentation was concentrated using Prostak microfiltration system (Millipore, USA) and further centrifuged at 6000 rpm for 30 min at 4 °C to recover cell pellet. The resulting cell pellet was either stored at –20 °C or processed for further purification. The process used for pilot scale purification was similar to the small scale purification with minor modifications. Briefly the cell paste was washed with cell wash buffer (1:10 w/v). The cells were disrupted using agitator bead mill (Wiley A. Bachofen, Switzerland). The cell suspension was disrupted by passing it three times through bead mill at a flow rate of 50 ml/min in a 0.6 l container at 1 bar pressure and centrifuged at 10,000 rpm for 30 min at 4 °C to recover pellet. The cell pellet containing the IBs was washed with IBs wash buffers followed by centrifugation at 10,000 rpm for 40 min at 4 °C. Solubilization of IBs was achieved by a mechanical homogenizer (Kinematica AG, Switzerland) for about 20 min in solubilization buffer (1:20 w/v) and further centrifuged at 10,000 rpm for 40 min at 4 °C as well as filtered with 0.45 µm membrane filter (Millipore, USA). This clarified solution containing rDen2 DIII protein was used for further purifications. The pilot scale purification was performed on an Akta Pilot Chromatography system using quik scale chromatography column (70/55-Millipore, USA) loaded with 500 ml Nickel charged streamline chelating resin (GE Healthcare, Sweden). Equilibration, washing and elution steps were the same as the small scale purifications. The eluted fractions were analyzed by SDS-PAGE and fractions having majority of rDen2 DIII protein band were pooled. Refolding was carried out by concentration of pooled eluted protein fractions using a 5 kDa cut-off ultrafiltration cassette (Millipore) and buffer exchanged against diafiltration buffer. This purified and refolded rDen2 DIII protein was further used for biological activity determination.

### 2.10. Analytical methods

Growth of the cultures was followed by measuring the optical density at 600 nm (OD_600_) with a spectrophotometer (Thermo, USA), by measuring the wet cell weight, and by determination of the dry cell weight. Whenever necessary the samples were diluted to a final OD value of less than 0.5. For wet cell weight measurement, 10 ml of cell suspension was centrifuged in pre-weighed 15 ml tubes at 8,000 rpm for 30 min. After decanting the liquid, pellets were measured for wet cell weight. For determination of dry cell weight, the pellets were dried at 105 °C for overnight. The protein concentration was determined spectrophotometrically at 280 nm using theoretical extinction coefficients or at 562 nm by the BCA methods. SDS-PAGE was carried out according to the method described elsewhere [24]. The 12% SDS-PAGE gel was used to check expressions and purity of rDen1 DIII and rDen2 DIII proteins. Proteins were stained with Coomassie Brilliant Blue or silver stain.

### 2.11. Immunization of mice

Female BALB/c mice of four to six weeks old were used for immunization. These mice were obtained from the animal house facility of our Institute. Immunogenicity of rDen1 DIII and rDen 2 DIII proteins with adjuvants were evaluated in BALB/c female mice. The techniques used for bleeding, and sacrifice of animals were strictly performed following mandates approved by the animal ethics committee (Committee for the Purpose of Control and Supervision of Experiments on Animals, Govt. of India) vide registration number 37/1999/CPCSEA. Groups of six mice were immunized subcutaneously with dose of 20–25 µg per mice of rDen1 DIII and rDen2 DIII proteins with Freund complete/incomplete adjuvant. Another group of six mice were immunized with rDen1 DIII and rDen2 DIII protein with alum. For control sera, mice were immunized with adjuvants and phosphate buffer saline (PBS). About 200 µl volume was used per mouse at each immunization. Priming was followed by two booster immunizations on days 21 and 42. Sera from immunized mice were collected on days 56.

### 2.12. Recombinant dengue virus type 1 and 2 specific antibodies

Titers of anti-rDen1 DIII and rDen2 DIII antibodies were determined by enzyme-linked immunosorbent assay similar to the method described earlier [9],[23] with minor modifications. Briefly, the microtiter ELISA plates were coated with rDen1 DIII and rDen2 DIII proteins (0.4 µg/100 µl/well) separately for 1 h at 37 °C in coating buffer (0.05 M carbonate buffer, pH 9.6). The plates were washed thrice with wash buffer (PBS containing 0.05% tween 20). The plates were then blocked with 200 µl of PBS containing 5% bovine serum albumin (BSA) for overnight at 4 °C. Test sera serially diluted (two fold) in PBS starting from 1:1,000 to 1:2,56,000 were incubated in triplicate wells (100 µl/well) at 37 °C for 1 h. The wells were washed three times with wash buffer. Hundred microliter of 1: 2,500 dilution of anti-mouse (IgG) antibodies conjugated to horseradish peroxidase (HRP) in PBS with 2% BSA were added to wells and incubated at 37 °C for 1 h. The wells were again washed as above and the plates were incubated at room temperature with 100 µl per well of the substrate solution containing o-phenylenediamine dihydrochloride and hydrogen peroxide at room temperature for 5–10 minutes. The reaction was stopped by 2.0 N sulphuric acid and the optical density (absorbance) was read at 490 nm in an ELISA reader (Biotek, USA).

### 2.13. Antibody isotyping

Antibody isotyping was carried out using method described elsewhere [25] using isotyping kit (Sigma, USA) with minor modifications. Microtiter ELISA plates were coated with rDen1 DIII and rDen2 DIII proteins (20 µg/100 µl/well) separately in coating buffer (carbonate-bicarbonate buffer) for 1 h at 37 °C. Plates were washed with PBST and blocked with PBS containing 5% BSA for overnight at 4 °C. The plates were again washed and 100 µl of mice sera diluted in PBS (1:200) were added in wells. The plates were incubated for 1 h at 37 °C. Primary antibody was reacted with goat anti-mouse IgG1, IgG2a, IgG2b and IgG3 isotype specific antibodies with 1:1,000 dilution at 37 °C for 1 h in triplicate. Detection of bound isotype specific antibodies was carried out with HRP labeled rabbit anti-goat IgG (1:5,000) and OPD substrate. The absorbance value was measured at 490 nm.

## 3. Results

### 3.1. Recombinant dengue virus type 1 and 2 domain III proteins

**Figure 1.**
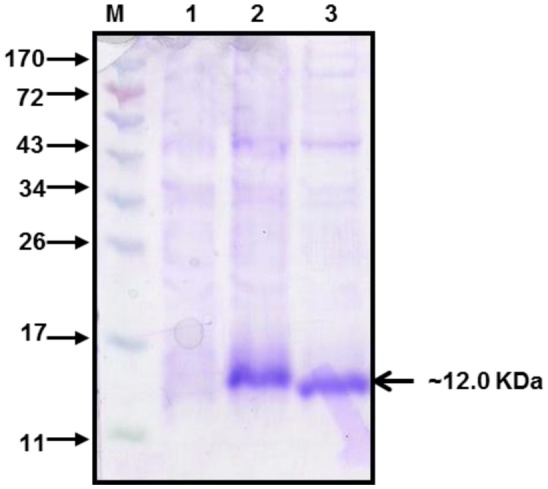
Expression profile of rDen1 DIII and rDen2 DIII proteins. Cells were lysed to recover inclusion bodies. Inclusion bodies were solubilized in solubilization buffer and centrifuged. The supernatant was subjected to 12% SDS-PAGE and gel was stained by coomassie brilliant blue. The protein band of ∼12 kDa confirmed the presence of the rDen1 DIII and rDen2 DIII proteins. Lane M, Molecular Weight Marker (kDa); lane 1, Un-induced culture; lane 2, rDen1 DIII protein; lane 3, rDen2 DIII protein.

The domain III genes of dengue virus type 1 and 2 were used for the expression of rDen1 DIII and rDen2 DIII proteins in *E. coli* respectively. The rDen1 DIII and rDen2 DIII genes were inserted into *Nde*I and *Sal*I sites and the resultant *Nde*I-*Sal*I fragments were cloned downstream of T7 promoter of *E. coli* expression vector pET30a+ to yield plasmid pET-Den1 DIII and pET-Den2 DIII separately. These pET30a + Den1 DIII and pET30a + Den2 DIII vector constructs are predicted to encode both of the recombinant proteins of ∼12 kDa in size. Both of the proteins were expressed as fusion to a C-terminal 6× histidine tag to aid purification. Recombinant plasmid was transformed with *E. coli* BLR(DE3) cells for expression of proteins. The expression profile of rDen1 DIII and rDen2 DIII proteins are shown in Figure 1.

### 3.2. Laboratory scale production and purification of rDen1 DIII and rDen2 DIII proteins

**Figure 2.**
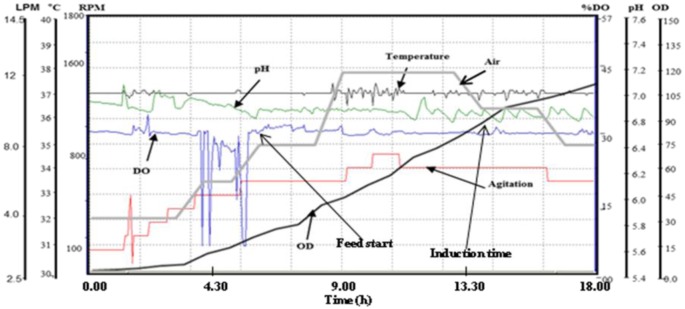
Real time profile of lab scale fed-batch fermentation process for the expression of recombinant dengue virus type 2 DIII protein. Cultivation was carried out in modified SB medium and culture was induced with IPTG (1 mM) at DCW of ∼16 g/l for rDen2 DIII protein expression. Figure shows the time profile for agitation (RPM), pH, temperature (°C), DO concentration (%), air ﬂow rate (liter per minute), and OD_600._

For laboratory scale production of rDen1 DIII and rDen2 DIII proteins, shake flask cultures, batch fermentations at 5 liter scale as well as fed-batch fermentations at 10 liter scale are employed in the present study. At shake flask culture using commonly employed LB medium, dry cell weight was 0.80 g/l and 0.81 g/l for the expression of rDen1 DIII and rDen2 DIII proteins respectively. However, OD at induction time was 0.6 for rDen1 and rDen2 DIII proteins using LB medium. Further cultivations using modified SB medium resulted dry cell weight of 2.56 g/l for rDen1 DIII protein and 2.60 g/l for rDen2 DIII protein. However, OD at induction time was 1.0 for rDen1 and rDen2 DIII proteins using modified SB medium. Both of the proteins were also produced using 5 liter fermentor under batch mode. For rDen2 DIII protein production, the DCW at induction and harvest times were 3.72 g/l and 7.10 g/l respectively. However, the DCW at induction and harvest times were 2.4 g/l and 5.0 g/l respectively for rDen1 DIII protein production. This may be attributed due to the difference in time course of batch fermentation processes for rDen1 DIII protein (8.5 h) and rDen2 DIII protein (9.5 h). Further cultivations using 10 l bioreactor under fed-batch mode, the DCW at induction and harvest times were about 12.0 g/l and 16.0 g/l respectively for rDen1 DIII protein. In case of rDen 2 DIII protein production using fed-batch mode, the DCW at induction and harvest times were 16.0 g/l and 21.80 g/l respectively. The fed-batch processes lasted for 14 hours in case of rDen1 DIII protein and 18 hours for rDen2 DIII protein production. The real time profile of fed-batch fermentation process for rDen2 DIII protein is shown in Figure 2.

**Figure 3.**
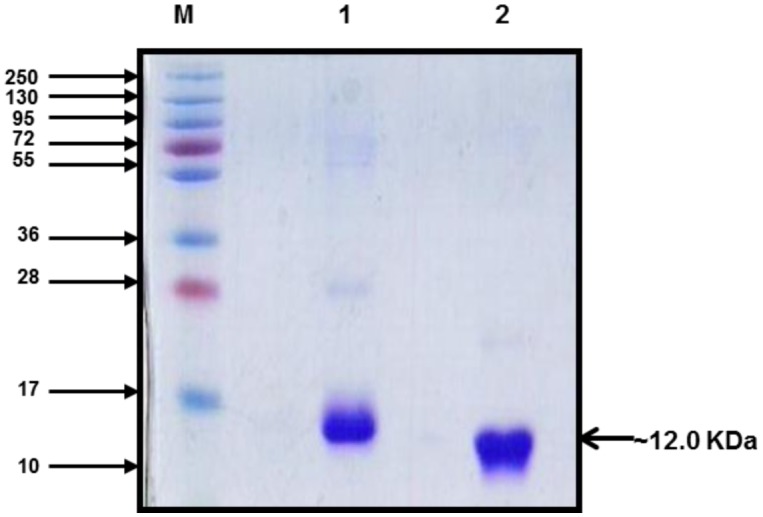
SDS-PAGE (Coomassie stained) profile of Immobilized metal affinity chromatography purified rDen1 DIII and rDen2 DIII proteins. The protein band of ∼12 kDa size confirmed the presence of the rDen1 DIII and rDen2 DIII proteins. Lane M, MW Marker (kDa); lane 1, rDen1 DIII protein; lane 2, rDen2 DIII protein.

The biomass recovered from shake flask cultures, batch fermentation and fed-batch fermentation processes were subjected to purifications to recover pure proteins. The IBs were solubilized and further processed for laboratory scale purification using Immobilized metal affinity chromatography with Hisprep 16/10 column on Akta Explorer FPLC system. The rDen1 DIII and rDen2 DIII proteins could bind separately onto the resin and the impurity in protein could be washed by lowering the pH of the buffer. The proteins could be eluted with elution buffer with pH 4.3. The purity of eluted proteins was up to 95% with SDS-PAGE analysis (Figure 3). The affinity purified rDen1 DIII and rDen2 DIII proteins were further diafiltered to get refolded and biologically active proteins. The rDen1 DIII and rDen2 DIII proteins yield after affinity purification were 11 mg/l and 60 mg/l respectively using LB medium. However, rDen1 DIII and rDen2 DIII proteins yield were 16 mg/l and 116 mg/l using modified SB medium.

Laboratory batch cultivation process using modified SB medium resulted in 62 mg/l of rDen1 DIII protein and 331 mg/l of rDen2 DIII protein after affinity chromatography. The rDen1 DIII protein and rDen2 DIII protein yield after fed-batch fermentation process was 161 mg/l and 1040 mg/l respectively. Up to two and three fold increase in the expression of both the proteins were observed for fed-batch processes in comparison with batch processes respectively. The protein yield after fed batch process using modified SB medium was fourteen fold higher for rDen1 DIII protein and seventeen fold higher for rDen2 DIII protein in comparison with shake flasks using commonly used LB medium. The dry cell weight and rDen1 DIII protein yield after batch and fed-batch cultivations are shown in Table 1.

The dry cell weight and rDen2 DIII protein yield after batch and fed-batch cultivations are shown in Table 2. Reproducibility of all the shake flask cultures, batch fermentations as well as fed-batch fermentations were confirmed with additional experiments conducted under the above specified optimal conditions and the final yield was within less than 10% of the result shown in Table 1 and 2. The dry cell weight of 22.25 g/l was obtained with additional fed-batch run carried out for rDen 2 DIII protein.

**Table 1.** Production characteristics of rDen 1 DIII protein using modified SB medium in *E. coli.*

Cultivation mode	Dry cell weight (g/l)	Total protein concentration (g/l)	Final product concentration (mg/l)	Specific product yield (mg/g)
Shake flask	2.56	0.657	16	6.26
Batch fermentation^a^	5.0	1.34	62	12.40
Fed-batch fermentation^b^	16.0	3.38	161	10.06

^a^At 5 liter scale; ^b^At 10 liter scale.

**Table 2.** Production characteristics of rDen 2 DIII protein using modified SB medium in *E. coli.*

Cultivation mode	Dry cell weight (g/l)	Total protein concentration (g/l)	Final product concentration (mg/l)	Specific product yield (mg/g)
Shake flask	2.60	0.628	116	44.61
Batch fermentation^a^	7.10	1.50	331	46.61
Fed-batch fermentation^b^	21.80	6.68	1040	47.70
Batch fermentation^c^	7.04	1.43	287	40.76

^a^At 5 liter scale; ^b^At 10 liter scale; ^c^At 100 liter scale.

### 3.3. Pilot Scale production and purification of rDen2 DIII protein

**Figure 4.**
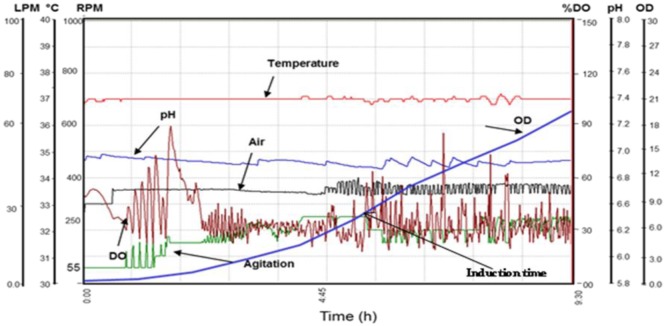
Real time profile of pilot scale batch fermentation process for the large scale expression of recombinant dengue virus type 2 DIII protein. Cultivation was carried out in modified SB medium and culture was induced with IPTG (1 mM) at DCW of ∼3.74 g/l for rDen2 DIII protein expression. Figure shows the time profile for agitation (RPM), pH, temperature (°C), DO concentration (%), air ﬂow rate (liter per minute), and OD_600._

To conduct vaccine studies with pure, refolded and biologically active recombinant proteins, large amount of biomass is necessary for purifications. Before the pilot scale fermentation, the cultivation conditions and culture medium were optimized at the small scale using shake flask culture and 5.0 liter fermentor. Under the cultivation conditions optimized at the small scale, pilot scale fermentation (100 liter) was carried out using modified SB medium to obtain large amount of biomass for rDen2 DIII protein. The real time profile of pilot scale batch fermentation process is shown in Figure 4.

**Figure 5.**
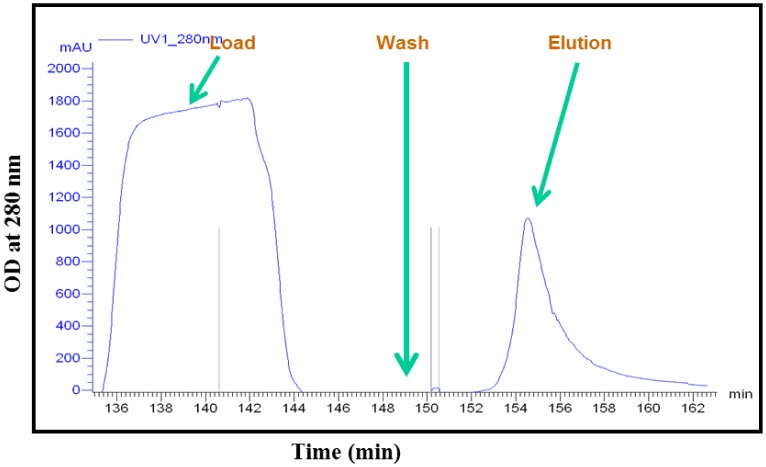
Chromatogram showing pilot scale purification of rDen2 DIII protein using Immobilized metal affinity affinity chromatography. The solubilized IBs containing rDen2 DIII protein was applied onto a pilot scale chromatography column (2 liter capacity Quik Scale column) containing 500 ml charged streamline chelating affinity chromatography resin. Unbound proteins were removed by washing the column with wash buffer (ten column volume) and purified protein was eluted with elution buffer.

Further pilot scale purification was done to get pure rDen2 DIII protein. The purified protein was diafiltered to recover biologically active protein for vaccine development studies. The dry cell weight at induction and harvest times were 3.74 g/l and 7.04 g/l respectively. For pilot scale purification of rDen2 DIII protein, a total of 500 ml of clear solubilized solution was loaded onto Quikscale column containing 500 ml of Ni charged streamline chelating resin. At the end of this purification step, the product concentration was 287 mg/l of culture. The affinity purified protein was refolded and this refolded protein was used for mice immunization. The successful production and purification of rDen2 DIII protein at pilot scale was initiated to foster further research with dengue vaccine development. The pilot fermentation resulted approximately 2.32 kg cell paste that was stored at −20 °C. The growth profile of pilot scale batch fermentation process is shown in Figure 4. The biomass and recombinant protein yield after pilot scale fermentation process is given in Table 2. Reproducibility of this pilot scale was confirmed with additional experiment conducted under the similar conditions and the final dry cell weight was 7.12 g/l. The chromatogram of pilot scale purification for rDen 2 DIII protein is shown in Figure 5.

### 3.4. Characterization of rDen1 DIII and rDen2 DIII proteins

**Figure 6.**
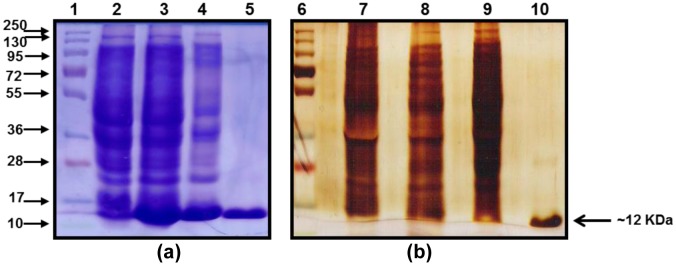
Characterization of pilot scale purified rDen2 DIII protein produced at 100 liter scale. (a) SDS-PAGE (Coomassie stained) profile of rDen2 DIII protein. Lane 1, MW Marker (kDa); Lane 2, Un-induced culture; lane 3, Lysate; lane 4, Load; Lane 5, Purified protein. (b) Purity of rDen2 DIII protein was assessed on silver stained SDS-PAGE gel. Lane 6, Molecular Weight Marker (kDa); Lane 7, Un-induced culture; lane 8, Lysate; lane 9, Load; lane 10, Purified protein.

The expression profile of rDen1 DIII and rDen2 DIII proteins were analyzed by SDS-PAGE and shown in Figure 1. Purified rDen1 DIII and rDen2 DIII proteins produced using laboratory scale batch cultivations were characterized for its purity (Figure 3). SDS-PAGE analysis of both the proteins resulted in protein band of about ∼12 kDa (Figure 3). Further characterization of pilot scale affinity chromatography purified rDen2 DIII protein was also performed using silver stained SDS-PAGE. This analyses demonstrated that the protein was highly pure (Figure 6). The SDS-PAGE gel analysis showed that more than 95% purity has been achieved using single step affinity chromatography (Figure 6).

### 3.5. Humoral response in mice immunized with recombinant dengue virus type 1 and 2 DIII proteins

**Figure 7.**
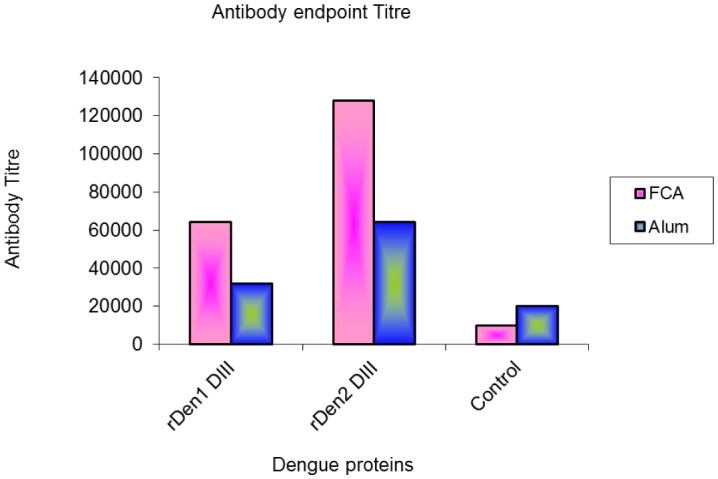
Endpoint titers for recognition of rDen1 DIII and rDen2 DIII proteins. Sera collected from mice immunized with protein and adjuvant formulations were tested for recognition of both proteins at various dilutions by ELISA.

Purified and refolded proteins of both the type of dengue virus were formulated with adjuvants and used to immunize mice. The rDen1 DIII and rDen2 DIII antigen with Freund's adjuvant developed higher levels of rDen1 DIII and rDen2 DIII specific antibodies compared to other groups as well as control group (Figure 7).

**Figure 8.**
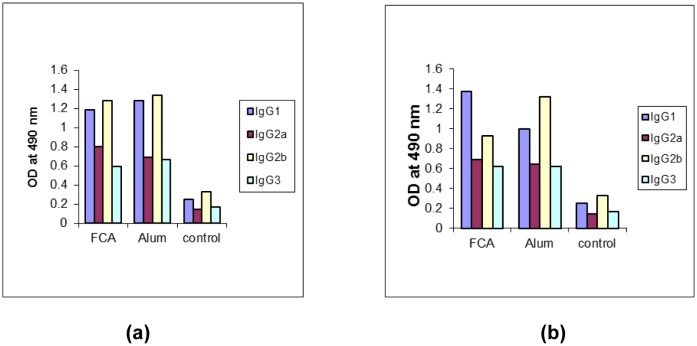
Profile of IgG subclass in mice sera. Sera samples collected on day 56 were analyzed for presence of rDen1 DIII and rDen2 DIII specific IgG1, IgG2a IgG2b and IgG3 subclass antibodies by ELISA. (a) For recombinant dengue virus type 1 domain III protein; (b) For recombinant dengue virus type 2 domain III protein.

Pre-immunization sera and adjuvant control sera were used as control. Sera collected from mice immunized with rDen1 and rDen2 DIII proteins in formulation with adjuvants were further analyzed for presence of different IgG sub classes (Figure 8a, 8b). These formulations induced predominantly elevated level of IgG1 and IgG2b isotypes as compared to IgG2a and IgG3.

## 4. Discussion

For development of vaccines against any disease, selection of antigen or protein is an important aspect. Envelope domain III protein of the Dengue virus is a major target antigen for virus neutralizing antibody responses. In the earlier studies, various investigators have expressed the domain III protein of dengue viruses in *E. coli*, insect cells or *Pichia pastoris* and evaluated the protection studies for vaccine development or diagnostic potential [3],[6],[7],[9],[10]. In our earlier report, diagnostic potential of recombinant dengue virus type 2 domain III protein was evaluated using small scale produced rDen2 DIII protein [26]. Production of heterologous protein in *E. coli* has been highly successful in recent past because it allows cost effective production of recombinant proteins in large amounts [11],[12],[14],[27]. To enhance the expression of recombinant dengue virus type 1 and 2 domain III proteins, LB and modified SB media were evaluated at shake flask cultures. The resultant biomass and protein yield was higher with modified SB medium in comparison with LB medium for both the proteins. Similar observations were also reported for the expression of other proteins using nutrient enriched medium [28],[29]. Thus, modified SB medium was used for further cultivations. Higher biomass as well as protein expression levels attained with modified SB medium can be explained, as this medium is rich in glycerol, yeast extract and phosphate salts.

The scale up of recombinant proteins is necessary because large amount of purified proteins are used for vaccine development studies [14],[30]. Lab scale batch and fed-batch cultivations were performed using modified SB medium for rDen1 DIII and rDen2 DIII proteins. In feed medium, glycerol and yeast extract was used as carbon source and nitrogen source respectively. The yeast extract present in media enhance the specific cellular yield of the expressed protein particularly during high cell density fermentation where the demand of nitrogenous source becomes very high following induction [14],[28]. The final dry cell weight after fed-batch process was found to be increased more than twenty times for rDen1 DIII protein and twenty six times for rDen2 DIII protein as compared to shake flask cultivations using LB medium.

Process development is the technological foundation that underlies the manufacture of new vaccines and is central to successful commercialization [31]. We have also produced rDen 2 DIII protein using pilot scale fermentor in modified SB medium. By using glycerol as carbon source in growth medium, large amount of biomass may be achieved [28],[29]. The phosphate salts present in the growth media provided a buffering capacity to prevent pH fluctuations that could adversely affect normal metabolism [32],[33]. At 5 liter scale, to maintain the DO concentration about 20–30%, the agitation varied from 200 to 500 rpm and aeration varied from 0.5 to 2.0 vvm. Further, at 100 liter scale, to keep the DO level identical with 5 liter scale, the aeration varied from 0.25 to 0.6 vvm and agitation varied from 50 to 350 rpm. The constant DO concentration at both the scales was used as scale up criteria for the present study. The biomass resulted from the pilot scale batch process was identical with lab scale batch process for rDen2 DIII protein (Table 2). Using similar strategy as used for rDen2 DIII protein at pilot scale, rDen1 DIII protein can also be produced at pilot scale.

We have also developed lab scale and pilot scale purification process for rDen1 DIII and rDen2 DIII protein respectively. The SDS-PAGE profile of purified proteins depicted clear band of rDen1 DIII and rDen2 DIII proteins with ∼12 kDa size (Figure 3 and Figure 6). The rDen2 DIII protein yield after lab scale purification process was 331 mg/l (Table 2). However, pilot scale purification process resulted 287 mg/l of rDen2 DIII protein (Table 2). The lower yield at pilot scale purification process may be attributed to the complexity associated with pilot scale as compared with small scale. Fed-batch processes resulted significant enhancement of rDen1 DIII (161 mg/l) and rDen2 DIII (1040 mg/l) proteins yield as compared to shake flasks (16 mg/l and 116 mg/l) as well as batch cultivations (62 mg/l and 331 mg/l). Earlier studies reported that the rDen2 DIII protein yield using *Pichia pastoris* expression system was 125 mg with 94% purity from 10 liter bioreactor culture [34]. The dengue tetravalent DIII protein yield using insect cells expression system was 0.3 mg per 10^6^ cells with 95% purity [10]. In *E. coli* expression system, higher product yield could be achieved in less time with minimum cost as compared to other expression systems like Yeast such as *Pichia pastoris* and mammalian expression system [11],[19]. This is the first report on the pilot scale purification of recombinant dengue virus type 2 domain III protein.

Various researchers studied the immunogenicity and protective efficacy of domain III proteins of one, two or all the serotypes of dengue viruses*. E. coli* expressed and purified Dengue-1 envelope protein domain III formulated with PELC plus CpG oligodeoxynucleotides enhances immune response and induced neutralizing antibodies against dengue-1 virus [3]. Purified tetravalent formulations containing domain III-P64k recombinant proteins of dengue virus types 1, 3 and 4 with the domain III-capsid protein from dengue virus type 2 which was expressed in *E. coli* resulted in neutralizing antibody response [6]. *E. coli* expressed and purified envelope domain III-capsid chimeric proteins of the four dengue serotypes as a tetravalent dengue vaccine candidate were evaluated and found to be able to generate humoral and cellular immunity [7]. Purified domain III protein of dengue virus type 4 which was expressed in *E. coli*, in combination with compatible adjuvants were evaluated and found to be highly immunogenic and elicited high titer neutralizing antibodies as well as cell mediated immune response [9]. A tetravalent protein by joining the receptor-binding envelope domain III (EDIII) of the four dengue virus serotypes was expressed using baculovirus expression system and purified for use in the diagnosis of dengue virus infections [10]. Semi-purified domain III-capsid chimeric protein of Dengue virus type 2 expressed in *E. coli* was also able to induce both humoral and cell-mediated immunity [35]. However, all these proteins used for vaccine studies were produced at small scale using shake flask cultures. For further trials studies in vaccine development, large amount of proteins are required, which could be bulk produced using bioreactors. In the present study, we have developed processes for bulk production of domain III proteins of dengue virus type 1 and 2 using 10 liters bioreactor. Further, we have also developed a pilot scale process using 100 liter bioreactor for production of domain III protein of dengue virus type 2. Pilot scale purification process was also developed for large scale purification of domain III protein of dengue virus type 2. In the present study, we have used domain III proteins of dengue virus type 1 and 2 produced at large scale for vaccine development studies.

Here, immunomodulatory potential of purified and renatured rDen 1 DIII and rDen2 DIII proteins in combination with adjuvants were studied in mice. Humoral responses were evaluated in mice and characterized by high titers of IgG antibody. In the previous report, the neutralizing antibody titers of recombinant dengue virus type 4 domain III protein have been evaluated by plaque reduction neutralization assay that showed the potential of this protein for virus neutralization [9]. Sera from mice immunized with both the proteins in combination of Freunds adjuvant resulted in highest ELISA titers in comparison to other formulation. Sera collected from immunized mice were further tested for presence of different IgG subclasses. The IgG1 and IgG2b subtypes were found to be most prevalent. The rDen1 DIII and rDen2 DIII antigens in combination with Freund's adjuvants resulted high titers of IgG1 and IgG2b. The subclasses of IgG immunoglobulins such as IgG1, IgG2a, IgG2b, and IgG3 provide the immunity to most of the infectious agents. Thus, the rDen1 DIII and rDen2 DIII proteins in combination with adjuvants elicited immune response, which plays an important role in the protection of viral infections.

## 5. Conclusion

Large scale production and purification of recombinant dengue virus proteins have become important for research in the area of dengue vaccine development. This is particularly relevant for recombinant domain III proteins due to their good immunogenic properties and diagnostic potential. The scale up methodology described in this study allows producing large quantities of recombinant dengue virus type 1 and 2 domain III proteins with high yield and purity. Purified and renatured proteins resulted high titer antibody and induces immune response in mice immunized with combination of adjuvants. Finally, this study provides us with some insight into the possible development of dengue vaccine.
